# Transcriptomics data of a human *in vitro* model of non-alcoholic steatohepatitis exposed to elafibranor

**DOI:** 10.1016/j.dib.2019.104093

**Published:** 2019-06-03

**Authors:** Joost Boeckmans, Karolien Buyl, Alessandra Natale, Valerie Vandenbempt, Steven Branson, Veerle De Boe, Vera Rogiers, Joery De Kock, Robim M. Rodrigues, Tamara Vanhaecke

**Affiliations:** a Department of *In Vitro* Toxicology & Dermato-Cosmetology (IVTD), Faculty of Medicine and Pharmacy, Vrije Universiteit Brussel. Laarbeeklaan 103, 1090 Brussels, Belgium; b Department of Urology, UZ Brussel. Laarbeeklaan 101, 1090 Brussels, Belgium

**Keywords:** NASH, Elafibranor, *In vitro*, Transcriptomics

## Abstract

The present dataset contains the transcriptomic characterization of a novel *in vitro* model of non-alcoholic steatohepatitis (NASH) as well as its transcriptomics read-outs for the evaluation of elafibranor, a potential anti-NASH compound. We report whole genome microarray data (Affymetrix HG U133 plus 2.0) of human multipotent stem cell-derived hepatic cells (hSKP-HPC) exposed to mediators of NASH. These cells were exposed to lipogenic inducers (insulin, glucose, fatty acids) and pro-inflammatory factors (IL-1β, TNF-α, TGF-β) to trigger hepatocellular responses characteristic of NASH. In addition, to evaluate the anti-NASH features of elafibranor, a dual peroxisome proliferator-activated receptor (PPAR) agonist that currently is under investigation as a potential anti-NASH therapeutic, was tested this *in vitro* set-up.

This paper provides a detailed description of the microarray data as well as an indication of their value for evaluating cell signaling pathways (*e.g.* NFκB network) during the *in vitro* evaluation of anti-NASH compounds. Raw microarray data of different testing conditions were deposited as.CEL files in the Gene Expression Omnibus of NCBI with GEO Series accession number GSE126484. Further interpretation and discussion of these data can be found in the corresponding research article (DOI: 10.1016/j.phrs.2019.04.016) Boeckmans et al., 2019.

**Table:** Specifications table

Subject area	Pharmacology
More specific subject area	Preclinical drug development
Type of data	Figures and tables
How data was acquired	Affymetrix Human Genome U133 plus 2.0 array
Data format	Raw (.CEL) and normalized
Experimental factors	Human skin-derived precursors (hSKP) were differentiated towards hepatic cells (hSKP-HPC) as previously documented [2]. These cells were exposed for 24h to a cocktail of insulin (100 nM), glucose (4,5 mg/mL), sodium oleate (65 μM), palmitic acid (45 μM), tumor necrosis factor alpha (TNF-α) (50 ng/mL), interleukin-1 beta (IL-1β) (25 ng/mL) and transforming growth factor beta 1 (TGF-β1) (8 ng/mL). The obtained *in vitro* model was termed ‘hSKP-HPC NASH’ and was evaluated in the presence or absence of elafibranor (10 μM or 30 μM). Bovine serum albumin (BSA) and dimethyl sulfoxide (DMSO)-treated samples served as controls.
Experimental features	Total RNA was extracted from ‘hSKP-HPC’ control samples (n = 3), ‘hSKP-HPC NASH’ (n = 3), ‘hSKP-HPC NASH’ + elafibranor 10 μM (n = 3) and ‘hSKP-HPC NASH’ + elafibranor 30 μM (n = 3). Analyses were conducted using Robust Multichip Average (RMA) Express, Transcriptome Analysis Console (TAC) (version 4.0.025, Applied Biosystems) and Ingenuity Pathway Analysis (IPA) (version 43605602, Qiagen).
Data source location	Department of *In Vitro* Toxicology and Dermato-Cosmetology (IVTD), Vrije Universiteit Brussel (VUB), Brussels, Belgium.
Data accessibility	Raw data is available at the Gene Expression Omnibus (GEO) from NCBI (GSE126484, www.ncbi.nlm.nih.gov/geo/query/acc.cgi?acc=GSE126484).
Related research article	J. Boeckmans, K. Buyl, A. Natale, V. Vandenbempt, S. Branson, V. De Boe, V. Rogiers, J. De Kock, R.M. Rodrigues, T. Vanhaecke, Elafibranor restricts lipogenic and inflammatory responses in a human skin stem cell-derived model of NASH, Pharmacol. Res., 2019, In Press [1].

**Table:** 

**Value of the data** • Human-based *in vitro* models can contribute to the pharmacological investigation of NASH and the development of potential anti-NASH drugs [3]. • These transcriptomics data of a human skin stem cell-derived in vitro model for NASH, can be used for data mining when investigating NASH *in vitro*. They can also be utilized in comparative transcriptomics studies using other human-based datasets. • This is the first publicly available microarray dataset evaluating elafibranor using stem cell-derived hepatic cells.

## 1 Data

**Fig. 1:**
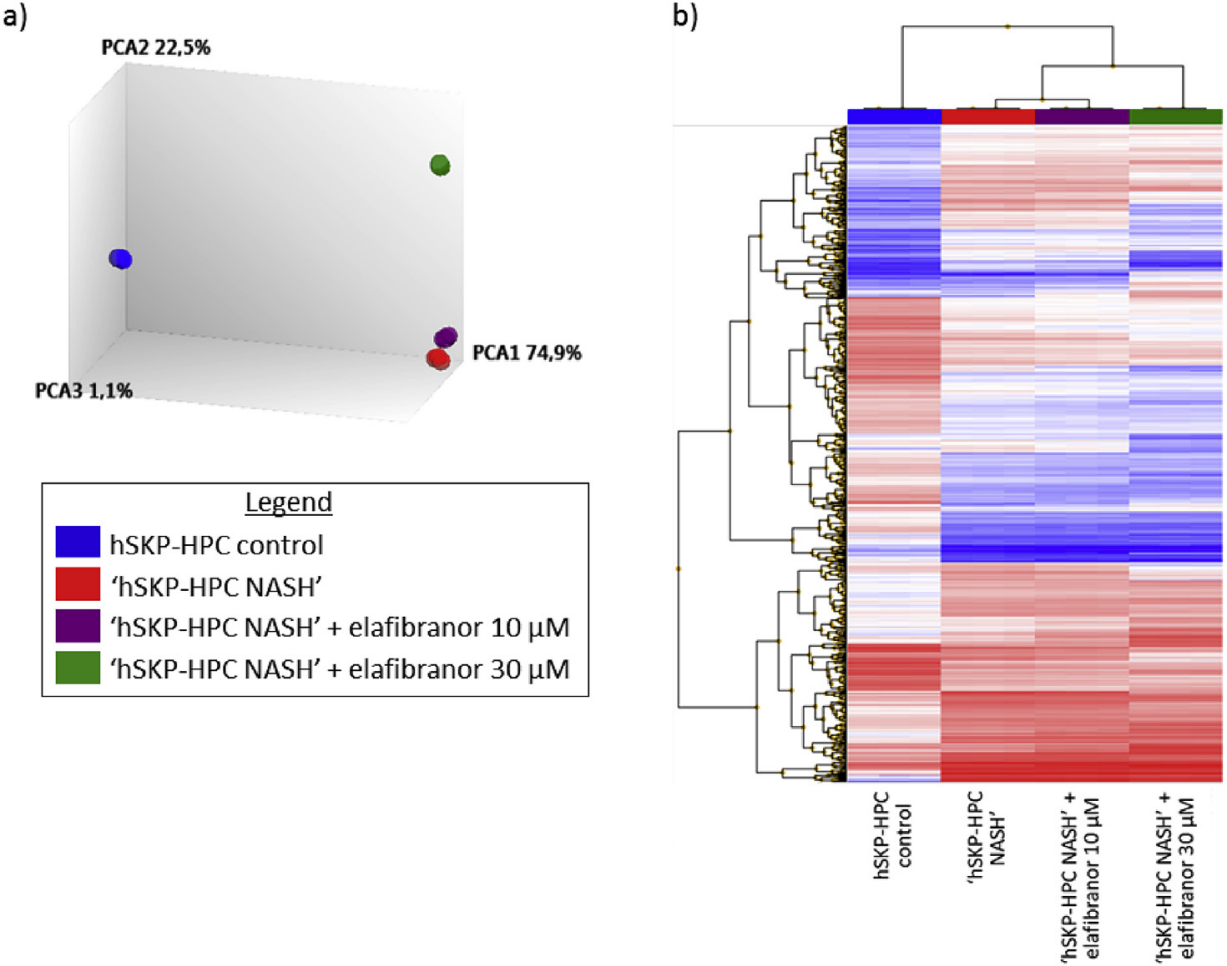
a) PCA plot of ‘hSKP-HPC control’ (n = 3), ‘hSKP-HPC NASH’ (n = 3), ‘hSKP-HPC NASH + 10 μM elafibranor (n = 3) and ‘hSKP-HPC NASH + 30 μM elafibranor (n = 3). b) Hierarchical clustering of ‘hSKP-HPC control’, ‘hSKP-HPC NASH’, ‘hSKP-HPC NASH’ + elafibranor 10 μM and ‘hSKP-HPC NASH’ + elafibranor 30 μM.

**Fig. 2:**
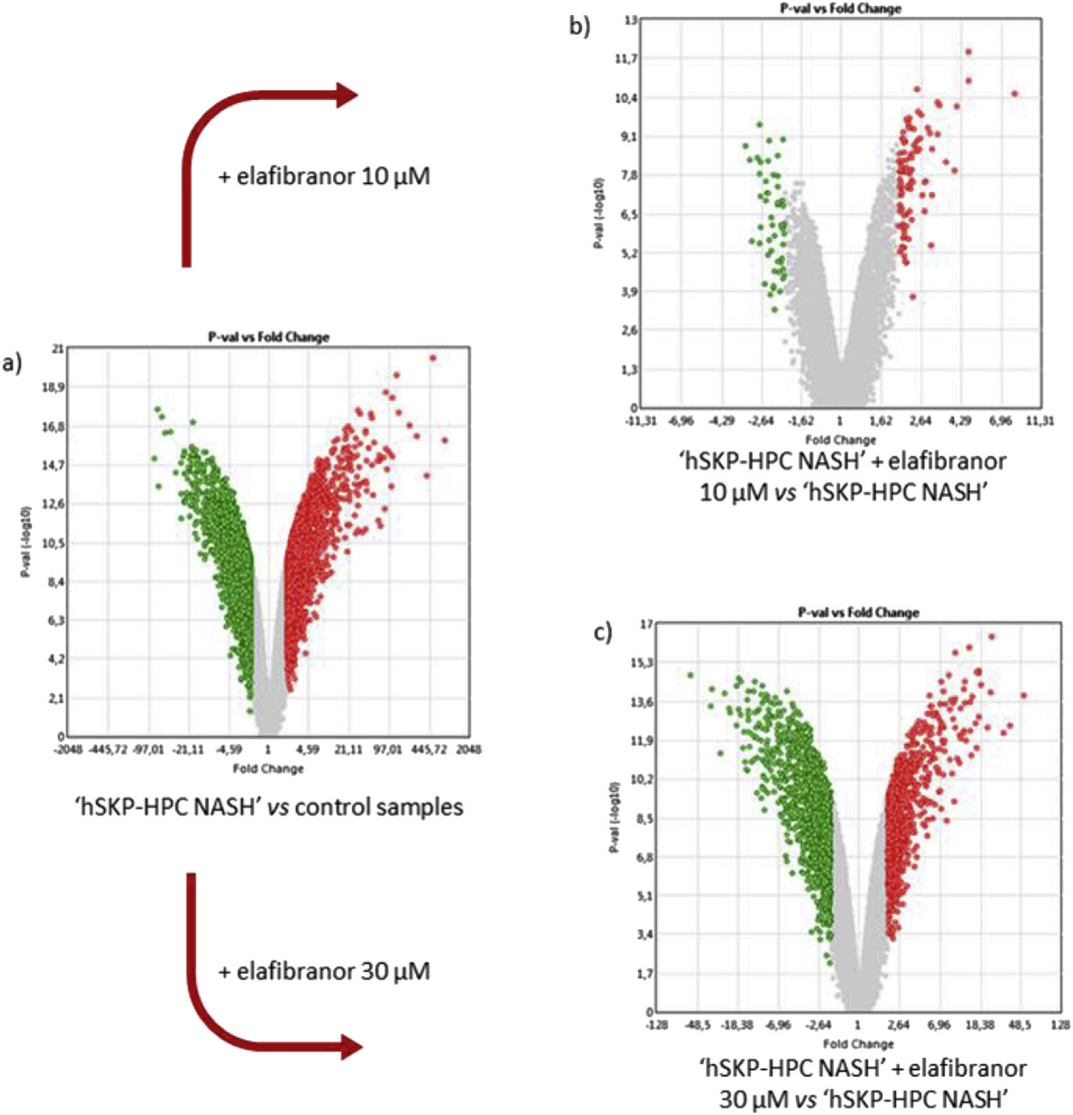
Volcano plots representing significantly modulated probesets between a) ‘hSKP-HPC NASH’ *vs* ‘hSKP-HPC’ control samples, (b) ‘hSKP-HPC NASH’ + elafibranor 10 μM *vs* ‘hSKP-HPC NASH’ and (c) ‘hSKP-HPC NASH’ + elafibranor 30 μM *vs* ‘hSKP-HPC NASH’. [Analysis cut-off: fold change [-2; +2], p < 0.05 (eBayes ANOVA)] [green = down-regulated; red = up-regulated].

**Fig. 3:**
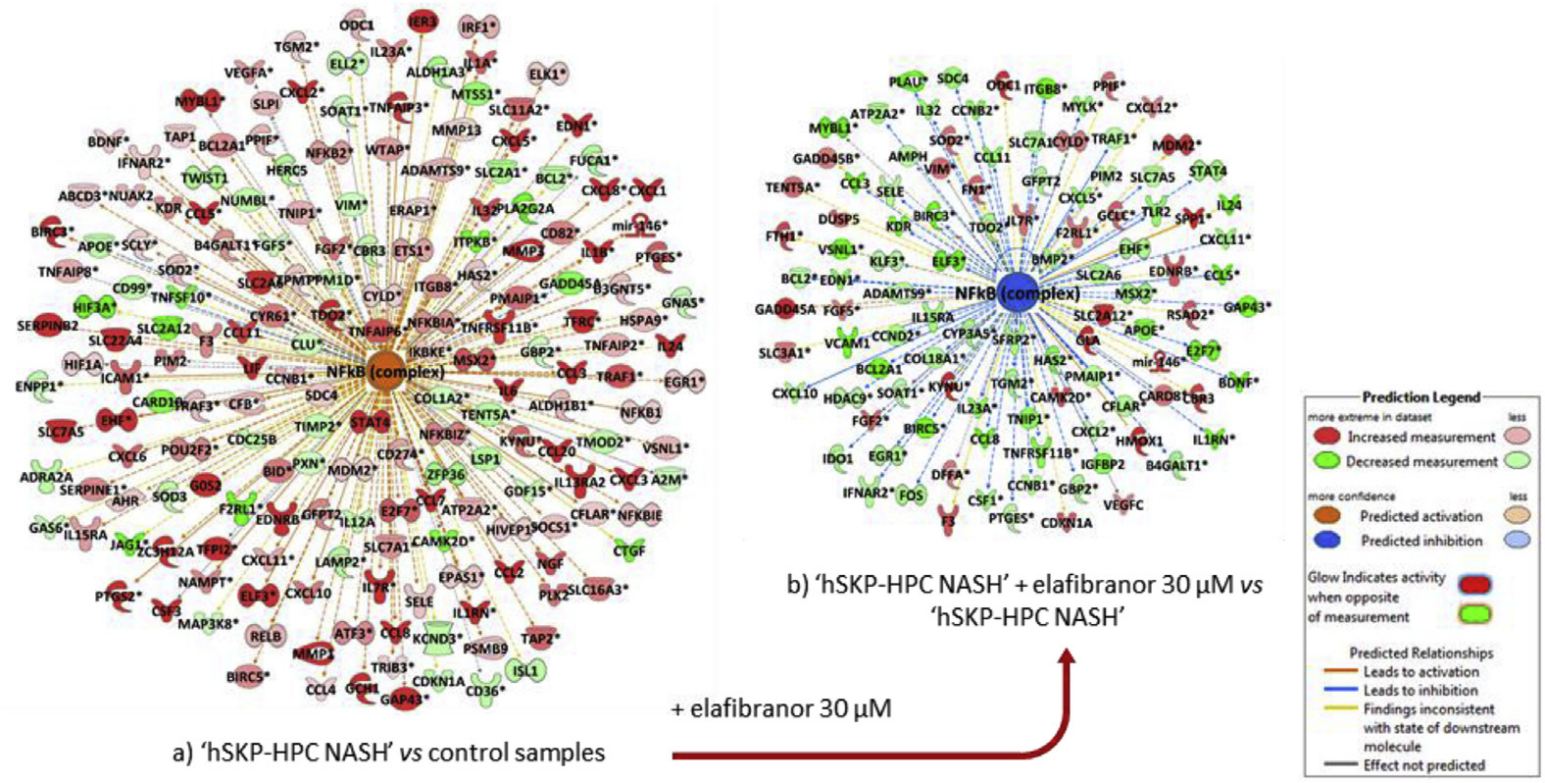
NFκB (complex) displayed as a network with a) ‘hSKP-HPC NASH’ *vs* control samples and b) ‘hSKP-HPC NASH’ + elafibranor 30 μM *vs* ‘hSKP-HPC NASH’. [Analysis cut-off: fold change [-2; +2], p ≤ 0.05 (Fischer's exact test)].

Whole genome transcriptomics data were obtained from hSKP-HPC exposed to a cocktail of insulin, glucose, fatty acids and inflammatory cytokines, mimicking NASH *in vivo.* In addition, data from NASH-triggered cells concomitantly exposed to elafibranor at two different concentrations is also reported. All data were generated using Affymetrix Human Genome U133 plus 2.0. and processed using Robust Multichip Average (RMA) Express, Transcriptome Analysis Console (TAC) (version 4.0.025, Applied Biosystems) and Ingenuity Pathway Analysis (IPA) (version 43605602, Qiagen). The transcriptomics data that were generated are visualized through a principle Component Analysis (PCA) plot (Fig. 1a), hierarchical clustering (Fig. 1b) and volcano plots (Fig. 2). Top 10 up- and down-regulated genes are listed in Table 1. A proof of principle of the use of the novel *in vitro* model for anti-NASH drug testing is represented in Fig. 3.

**Table 1:** Top-10 highest up- and down-regulated genes between ‘hSKP-HPC NASH’ *vs* control samples, ‘hSKP-HPC NASH’ + elafibranor 10 μM *vs* ‘hSKP-HPC NASH’ and ‘hSKP-HPC NASH’ + elafibranor 30 μM *vs* ‘hSKP-HPC NASH’. [Analysis cut-off: fold change [-2; +2], p ≤ 0.05 (Fischer's exact test)].

	‘hSKP-HPC NASH’ *vs* control samples		‘hSKP-HPC NASH’ + elafibranor 10 μM *vs* ‘hSKP-HPC NASH’		‘hSKP-HPC NASH’ + elafibranor 30 μM *vs* ‘hSKP-HPC NASH’	
	Gene	Fold change	Gene	Fold change	Gene	Fold change
Top upregulate	*CCL20*	649.2	*F2RL2*	7.8	*F2RL2*	49.1
	*CXCL5*	505.4	*SLC7A11*	4.7	*LINC00304*	38.5
	*CCL8*	388.5	*NMRAL2P*	4.2	*STYK1*	38.0
	*PTGS2*	178.5	*OSGIN2*	3.8	*HMOX1*	24.2
	*MMP3*	138.9	*CES1*	3.5	*MAP2*	19.0
	*CXCL8*	126.2	*SEL1L3*	3.0	*KCNE4*	18.1
	*C15orf48*	121.9	*NQO1*	3.0	*mir-146*	17.7
	*SERPINB4*	102.1	*PIR*	2.9	*NMRAL2P*	17.6
	*CSF3*	91.8	*TMEFF2*	2.9	*SLCO2B1*	17.2
	*HCK*	87.8	*MRPS15*	2.8	*HIST1H4F*	16.8
Top down-regulated	*SELENOP*	−77.3	*PI15*	−3.1	*CCL5*	−76.6
	*SLC40A1*	−70.9	*SLC38A1*	−3.0	*CCL19*	−37.6
	*AQP3*	−66.4	*CCL5*	−3.0	*DIRAS3*	−27.8
	*ST8SIA4*	−57.6	*VCAM1*	−2.9	*DTL*	−23.2
	*ADH1B*	−39.3	*TSPAN11*	−2.8	*GAP43*	−19.1
	*PPL*	−34.4	*PSAT1*	−2.7	*IL24*	−18.8
	*AHNAK2*	−31.1	*MEG8*	−2.7	*IL1RN*	−18.2
	*DEPP1*	−28.0	*KLHL24*	−2.6	*WISP1*	−17.3
	*STMN2*	−27.0	*MXRA5*	−2.6	*METTL7A*	−17.3
	*SYNE2*	−25.8	*RAB27B*	−2.5	*MXRA5*	−16.8

## 2 Experimental design, materials and methods

hSKP were differentiated towards hSKP-HPC according to an earlier established 24-day protocol [3]. Subsequently, hSKP-HPC were exposed to insulin (100 nM), glucose (4.5 mg/mL), sodium oleate (65 μM), palmitic acid (45 μM) (all purchased from Sigma-Aldrich), tumor necrosis factor-α (50 ng/mL) (Prospec), interleukin-1β (25 ng/mL) and transforming growth factor-1β (8 ng/mL) (both purchased from Peprotech). Bovine serum albumin (BSA) fatty acid free (Sigma-Aldrich) 7% (w/v) (Sigma-Aldrich) was used to complex sodium oleate in day-24-medium. Palmitic acid and elafibranor (Adooq Bioscience) were dissolved in dimethyl sulfoxide (DMSO) (Sigma-Aldrich). Final concentrations of BSA and DMSO in the exposing media were 1.4% (w/v) and 0.15% (v/v), respectively. Exposures were performed for 24h, in the presence or absence of elafibranor (10 μM and 30 μM).

RNA extractions and microarray procedures were performed according to De Kock *et al.* [4]. Three biological replicates of each condition were used. The PCA plot, hierarchical cluster and volcano plots were generated using TAC (RMA-normalized). Pathway analyses were conducted using IPA for which the data were prior normalized using RMA Express. PCA and hierarchical clustering of all analyzed samples are given in Fig. 1.

Differentially regulated probesets in ‘hSKP-HPC NASH’ *versus* untriggered hSKP-HPC, which correspond to 3173 differentially expressed genes, are shown in Fig. 2 a–c show the probesets that were significantly modulated in ‘hSKP-HPC NASH’ treated with elafibranor at 10 μM and 30 μM, respectively corresponding to 107 and 1667 differentially expressed genes.

The 10 highest up-regulated and down-regulated genes in ‘hSKP-HPC NASH’ *versus* control samples as well as the highest gene expression modulations induced by elafibranor are shown in Table 1.

To describe the value of the above described data in the elucidation of molecular mechanisms involved in the development or reduction of NASH, the activation of the NFκB pathway, which is a prototypical pro-inflammatory signaling pathway, has been investigated. As shown in Fig. 3, the NFκB complex is activated in the ‘hSKP-HPC NASH’ model, but becomes inhibited in the presence of elafibranor (30 μM). Further analysis of this finding as well as interpretation of the reported data in the context of evaluation of anti-NASH properties of elafibranor, can be found in the corresponding research article [1].
